# Development and validation of a biomarker index for HCC treatment response

**DOI:** 10.1097/HC9.0000000000000466

**Published:** 2024-06-19

**Authors:** Jeff Liang, Po-Yi Li, Joshua Norman, Marie Lauzon, Yee Hui Yeo, Hirsh Trivedi, Walid S. Ayoub, Alexander Kuo, Marc L. Friedman, Kamya Sankar, Jun Gong, Arsen Osipov, Andrew Hendifar, Tsuyoshi Todo, Irene Kim, Georgios Voidonikolas, Todd V. Brennan, Steven A. Wisel, Justin Steggarda, Kambiz Kosari, Rola Saouaf, Nicholas Nissen, Francis Yao, Neil Mehta, Ju Dong Yang

**Affiliations:** 1 Department of Internal Medicine, Cedars-Sinai Medical Center, Los Angeles, California, USA; 2 Department of Medicine, Division of Gastroenterology, University of California, San Francisco, San Francisco, California, USA; 3 Department of Internal Medicine, Stanford University, Palo Alto, California, USA; 4 Biostatistics and Bioinformatics Research Center, Cedars-Sinai Medical Center, Los Angeles, California, USA; 5 Department of Internal Medicine, Karsh Division of Gastroenterology and Hepatology, Cedars-Sinai Medical Center, Los Angeles, California, USA; 6 Comprehensive Transplant Center, Cedars-Sinai Medical Center, Los Angeles, California, USA; 7 Department of Radiology, Cedars-Sinai Medical Center, Los Angeles, California, USA; 8 Samuel Oschin Comprehensive Cancer Institute, Cedars-Sinai Medical Center, Los Angeles, California, USA; 9 Department of Surgery, Cedars-Sinai Medical Center, Los Angeles, California, USA; 10 Department of Surgery, Division of Transplant Surgery, University of California, San Francisco, San Francisco, California, USA

## Abstract

**Background:**

Serum AFP-**L**3%, **A**FP, and **D**CP are useful biomarkers for HCC detection, but their utility in assessing treatment response remains unknown. We aim to evaluate the accuracy of a biomarker model in the detection of posttreatment viable tumors.

**Methods:**

For model derivation, recipients with HCC undergoing liver transplant from 2018 to 2022 who had biomarkers collected within 3 months before transplant were included. We developed a generalized linear model for detecting posttreatment viable tumors with the 3 biomarkers as covariates, which we termed the “LAD Score.” An independent cohort of 117 patients with HCC was used for external validation.

**Results:**

Among 205 recipients of transplant, 70.2% had evidence of viable tumor on explant. The median LAD score was higher among patients with viable versus nonviable tumors (1.06 vs. 0.465, *p* < 0.001). The LAD score had a sensitivity of 55.6% and a specificity of 85.1% at the cutoff of 0.927, which was more accurate than imaging for detecting posttreatment viable tumors (AUROC 0.736 vs. 0.643, respectively; *p* = 0.045). The superior performance of the LAD score over imaging is primarily driven by its greater accuracy in detecting tumors <2 cm in diameter (AUROC of the LAD score 0.721 vs. imaging 0.595, *p* = 0.02). In the validation data set, the LAD score had an AUROC of 0.832 (95% CI: 0.753, 0.911) with a sensitivity of 72.5% and a specificity of 89.4% at the cutoff of 0.927.

**Conclusions:**

Our findings suggest the utility of LAD score in treatment response assessment after locoregional therapy for HCC, particularly in detecting small tumors. A larger prospective study is in progress to validate its accuracy and evaluate its performance in recurrence monitoring.

## Graphical abstract

**Figure:**
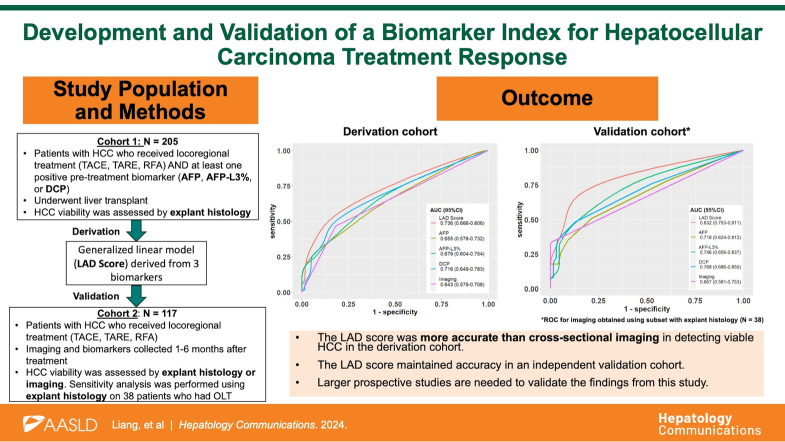

## INTRODUCTION

HCC is the sixth leading cause of cancer-related death worldwide and is most commonly seen in those with cirrhosis or chronic HBV.^1–3^ Routine HCC surveillance is a cost-effective approach that improves early-stage detection and survival, and current guidelines in the United States recommend screening all high-risk patients using transabdominal ultrasound and serum alpha-fetoprotein (AFP) every 6 months.^4–8^ However, ultrasound has limited sensitivity (estimated 40%–80%) due to operator technique and patient anatomy (ie, obesity and overlying bowel), and AFP has poor sensitivity for detecting small tumors and can be falsely elevated in chronic viral hepatitis and gonadal malignancy.^2,9^ Additional biomarkers, such as lens culinaris-agglutinin-reactive fraction of AFP (AFP-L3%) and des-gamma-carboxy prothrombin (DCP), have demonstrated predictive utility alone and in combination with AFP.^10–15^ The GALAD score, which was developed using **g**ender, **a**ge, AFP-**L**3%, **A**FP, and **D**CP, had a sensitivity and specificity of ~90% for detecting HCC and has been validated in multiple phase II studies.^15–21^

Few studies have examined the utility of biomarkers for surveillance after locoregional treatment. This represents an important area of study since chemoembolization is one of the most frequently used treatment modalities and radioembolization is increasingly used for HCC therapy. Surveillance imaging can be obscured by the posttreatment effect, and the current LI-RADS Treatment Response (LR-TR) radiologic criteria for detecting viable tumors has poor sensitivity (35%–54%) and may yield equivocal results.^22–24^ Tumor recurrence also occurs frequently in patients who undergo locoregional therapy, further emphasizing the need for accurate detection.^25–27^ We aimed to (1) evaluate the accuracy of HCC biomarkers in detecting tumor viability following locoregional cancer treatment, (2) develop and validate an HCC biomarker model to detect posttreatment viable tumors.

## METHODS

In this multi-institution retrospective cohort study, we identified patients at UCSF and Cedars-Sinai who had the HCC biomarker panel (AFP, AFP-L3%, and DCP) measured after locoregional treatments. Biomarker levels and basic demographics (age, sex, race/ethnicity, and etiology of chronic liver disease) were extracted from medical records. The UCSF database was used for model derivation, and the Cedars-Sinai database was used for model validation. This study was approved by the Cedars-Sinai and UCSF Institutional Review Board. All authors had access to the study data and reviewed and approved the final manuscript.

### Patient selection and variables

#### Derivation cohort

Patients with HCC who underwent a liver transplant at UCSF from 2018 to 2022 and had the biomarkers AFP, AFP-L3%, and DCP and cross-sectional imaging obtained within 3 months before liver transplant (and at least 1 month after the last cycle of locoregional treatment) were identified. Patients who take warfarin were excluded as it can falsely increase DCP levels in the absence of HCC (Supplemental Figure S1, http://links.lww.com/HC9/A927). Because biomarker-negative tumors before treatment are unlikely to turn positive after locoregional treatment, patients were subsequently excluded if all 3 biomarkers at the time of listing were negative for model derivation, defined as AFP <10 ng/mL, AFP-L3% <10%, and DCP <7.5 ng/mL.^28,29^ Biomarker levels and imaging results (number and size of viable tumors) at the time of listing and transplant, the number of locoregional treatments, and explant histology (viable tumor size, number, cumulative diameter, differentiation, and the presence of micro/macrovascular invasion) were recorded. Based on the pathology report of the viable tumors, patients’ tumor burden was categorized as being “within Milan criteria” (single tumor ≤5 cm OR ≤3 tumors with no tumor larger than 3 cm AND without extrahepatic or major vessel involvement) or “outside Milan criteria” (previous conditions not met). The assessment of pretransplant tumor viability was based on the LI-RADS CT/MRI treatment response algorithm.^30^ Individuals were classified as HCC positive or negative based on the presence or absence of viable tumors on explant histology.

#### Validation cohort

Patients with HCC who sought care at the Cedars-Sinai Medical Center from January 1, 2019, to June 1, 2023, and had AFP, AFP-L3%, and DCP measured were identified using the Deep6 AI software (deep6.cshs.org). Individuals were considered for inclusion if they (1) had cirrhosis or chronic hepatitis B infection, (2) were diagnosed with HCC and underwent locoregional therapy (including transarterial chemoembolization [TACE], transarterial radioembolization, or ablation), and (3) had at least 1 follow-up posttreatment surveillance imaging. Patients taking warfarin or had distant metastasis were excluded. Unlike with the derivation cohort, patients with pretreatment biomarker-negative HCC were included in the primary analysis. The first set of biomarkers obtained after treatment were reported; however, biomarker panels drawn <1 month after locoregional treatment were excluded to avoid falsely elevated results due to hepatic inflammation and injury in the acute posttreatment setting, and the subsequent panel was reported instead. Posttreatment tumor status was classified as “viable” or “nonviable” based on cross-sectional imaging (obtained between 1 and 6 months after treatment and within 3 months of biomarker measurement) according to the LI-RADS CT/MRI treatment response algorithm.^30^ Individuals who underwent liver transplants had tumor viability evaluated using explant histology, which was compared to imaging and biomarkers results obtained within 3 months before transplant. Subsequent images were used to determine the status of tumor viability in individuals with LR-TR equivocal response. Demographic and clinical information, including age at the time of biomarker collection, sex, race/ethnicity (obtained from the electronic health records), etiology of cirrhosis, tumor size/number at initial treatment, and treatment modality were also collected.

### Statistical analysis

The Fisher exact test was used to compare demographic and clinical categorical variables between the 2 cohorts, and a 2-tailed Wilcoxon rank sum test was used to compare continuous variables. In the derivation cohort, the difference in median biomarker levels between viable and nonviable tumors was calculated and compared using the Wilcoxon test. Log transform was applied to AFP and DCP levels to reduce skew and based on derivation from prior models, such as the GALAD score.^16^ A multivariate generalized linear model incorporating the 3 biomarkers was developed to predict the presence of HCC on explant. The AUROC curve was calculated, and the Youden index was used to determine the optimal cutoff of the multivariable model, which we term the “LAD” score (so named because we did not include the demographic variables **g**ender and **a**ge from the GALAD score, since the patients in our study cohort already have known HCC—while male gender and older age are associated with increased risk of HCC in the general population, they are not associated with treatment response). The accuracy of the LAD score in assessing HCC on explant was compared to that of cross-sectional imaging obtained within 3 months of transplant, and the Delong test was used to compare AUROC of the LAD score, AFP, and cross-sectional imaging. Sensitivity analysis was performed by comparing the sensitivity and specificity of the LAD score, individual biomarkers, and imaging when stratified by the etiology of HCC. In addition, the performance of the LAD score in imaging-negative cases was assessed in the derivation cohort. The accuracy of the biomarker model was evaluated using the validation cohort, and the sensitivity and specificity were reported using the optimal cutoff from the derivation set. Finally, an exploratory analysis was performed by developing a generalized linear model incorporating the 3 biomarkers and LR-TR viability on pretransplant cross-sectional imaging (viable = 1, nonviable = 0); we elected not to use this as the primary model due to the limited number of patients in the validation cohort with available histology data. All statistical analysis was performed using R software (version 4.2.2; R Foundation)^31^; the “pROC” and “ggplot2” packages were used for AUROC analysis and graphics design, respectively. A significance level of 0.05 was used for all analyses.

## RESULTS

### Derivation cohort

A total of 205 patients in the UCSF cohort were included for model derivation, after excluding 56 (21.5%) individuals with triple-negative pretreatment biomarkers (Table 1). Half of the patients were <65 years of age (52.7%) and more than two-thirds were male (72.2%). The most common race/ethnicity was non-Hispanic White (40.5%) followed by Hispanic (31.7%). HCV was the most common etiology of liver disease (49.8%) followed by metabolic dysfunction–associated steatohepatitis (MASH) and alcohol (14.1% for both). Patients on average underwent 2 episodes of locoregional treatment before liver transplant (SD: 1.65). There was no statistically significant difference in demographics, tumor size, or number of tumors between this cohort and the excluded biomarker-negative cohort (Supplemental Table S1, http://links.lww.com/HC9/A927).

**TABLE 1:** Clinical characteristics of patients in derivation versus validation data sets

Category	Derivation data set (N = 205)	Validation data set (N = 117)	*p*
Age at biomarker measurement, n (%)			**<0.001**
<65 y	108 (52.7)	39 (33.3)	
≥65 y	97 (47.3)	78 (66.7)	
Sex, n (%)			0.38
Male	148 (72.2)	79 (67.5)	
Female	57 (27.8)	38 (32.5)	
Race, n (%)			0.15
White (non-Hispanic)	83 (40.5)	39 (33.3)	
Hispanic	65 (31.7)	52 (44.4)	
Asian	41 (20.0)	19 (16.2)	
Black/other	16 (7.8)	7 (6.0)	
Etiology of cirrhosis, n (%)			0.12
Hepatitis C	102 (49.8)	42 (35.9)	
Metabolic dysfunction–associated steatohepatitis	29 (14.1)	26 (22.2)	
Alcohol	29 (14.1)	21 (17.9)	
Hepatitis B	24 (11.7)	17 (14.5)	
Other/unknown	21 (10.2)	11 (9.4)	
Pretreatment tumor burden^a^ (25–75th percentile)			
Median number of tumors	1 (1, 2)	1 (1, 2)	0.19
Median largest tumor size (cm)	2.5 (2.1, 3.3)	2.7 (2.0, 4.0)	0.46
Median biomarker value (25–75th percentile)			
AFP (ng/mL)	6.0 (3.0, 14.0)	5.7 (3.3, 14.7)	0.59
AFP-L3%	8.6 (0.5, 14.9)	7.5 (0.25, 12.7)	0.07
DCP (ng/mL)	1.0 (1.0, 3.2)	1.0 (1.0, 2.4)	0.27

Significant (*p* < 0.01) values are in bold.

a Pretreatment tumor burden is determined by cross-sectional imaging.

Abbreviations: AFP, alpha-fetoprotein; AFP-L3%, lens culinaris-agglutinin-reactive fraction of AFP; DCP, des-gamma-carboxy prothrombin.

### Validation cohort

A total of 117 patients were included for model validation. Patients were older as 66.7% of patients were ≥ 65 years of age at the time of biomarker collection, and 32.5% of patients were female. The most common etiology of cirrhosis was HCV (35.9%) followed by MASH (22.2%). One hundred three (88%) patients were evaluated for HCC using MRI; the remainder were evaluated with CT. Compared to the derivation cohort, a significantly greater proportion of patients were older than 65 years of age (Table 1). There were no significant differences in sex, race/ethnicity, initial tumor burden, and biomarker levels between the validation and derivation cohorts. TACE was the initial treatment modality in 47 (40.2%) patients, transarterial radioembolization in 45 (38.5%) cases, and ablation in 25 (21.4%) cases.

### LAD score development for posttreatment tumor detection

**FIGURE 1:**
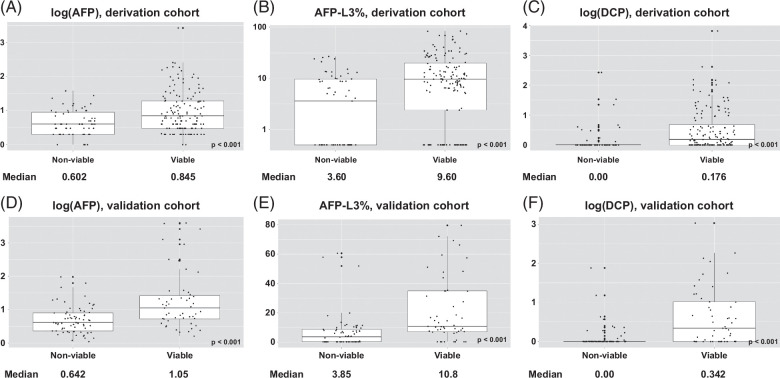
Comparison of serum biomarker levels among patients with viable tumors versus nonviable tumors in the derivation set and validation set. (A) Log (AFP), derivation set (bars represent 25th and 75th percentiles, vertical lines extending out from the box represent 1.5 [Quartile 3 – Quartile 1], bold line inside the box plot represents median levels). Level of significance: *p* < 0.001 (Wilcoxon test). (B) AFP-L3%, derivation set (bars represent 25th and 75th percentiles, vertical lines extending out from the box represent 1.5 [Quartile 3 − Quartile 1], bold line inside the box plot represents median levels). Level of significance: *p* < 0.001 (Wilcoxon test). (C) Log (DCP), derivation set (bars represent 25th and 75th percentiles, vertical lines extending out from the box represent 1.5 [Quartile 3 − Quartile 1], bold line inside the box plot represents median levels). Level of significance: *p* < 0.001 (Wilcoxon test). (D) Log (AFP), validation set (bars represent 25th and 75th percentiles, vertical lines extending out from the box represent 1.5 [Quartile 3 − Quartile 1], bold line inside the box plot represents median levels). Level of significance: *p* < 0.001 (Wilcoxon test). (E) AFP-L3%, validation set (bars represent 25th and 75th percentiles, vertical lines extending out from the box represent 1.5 [Quartile 3 − Quartile 1], bold line inside the box plot represents median levels). Level of significance: *p* < 0.001 (Wilcoxon test). (F) Log (DCP), validation set (bars represent 25th and 75th percentiles, vertical lines extending out from the box represent 1.5 [Quartile 3 − Quartile 1], bold line inside the box plot represents median levels). Level of significance: *p* < 0.001 (Wilcoxon test). Abbreviations: AFP, alpha-fetal protein; AFP-L3%, lens culinaris-agglutinin-reactive fraction of AFP; DCP, des-gamma-carboxy prothrombin.

A total of 144 out of the 205 patients in the derivation set (70.2%) had evidence of HCC on explant; among those with viable HCC, AFP was ≥10 in 39.6% of the cases. All biomarker levels were significantly higher in individuals with viable tumors compared to those with nonviable tumors (Figure 1). In the univariate generalized linear models, each biomarker was individually associated with an OR >1 for HCC (Table 2). All 3 markers were prespecified given their role in HCC detection and used in the multivariable model development and the final LAD score was developed with the following formula:


$$\mathrm{LAD Score}=-0.3695+0.6915\times \log\;\left(\mathrm{AFP}\right)+0.0414\;\times \mathrm{AFP}-L3\%+1.180\times \log\left(\mathrm{DCP}\right).$$

**TABLE 2:** Generalized linear models predicting HCC viability on explant histology

	Coefficient	OR (95% CI)	*p*
Univariate models			
Biomarker			
Log_10_AFP	1.36	3.89 (1.89, 8.00)	0.0002
AFP-L3%	0.07	1.07 (1.03, 1.12)	0.0004
Log_10_ DCP	1.48	4.40 (1.81, 10.69)	0.001
Multivariable model (3 biomarkers)			
Predictor			
Intercept	−0.37	0.69 (0.37, 1.31)	0.26
Log_10_AFP	0.69	2.00 (0.84, 4.72)	0.12
AFP-L3%	0.04	1.04 (1.00, 1.09)	0.06
Log_10_ DCP	1.18	3.26 (1.31, 8.08)	0.01
Multivariable model (3 biomarkers and imaging)			
Predictor			
Intercept	−0.74	0.48 (0.23, 0.94)	0.04
Log_10_AFP	0.72	2.05 (0.89, 5.07)	0.10
AFP-L3%	0.04	1.04 (1.00, 1.09)	0.07
Log_10_ DCP	1.06	2.90 (1.27, 8.11)	0.02
Imaging	1.26	3.53 (1.68, 7.93)	0.001

Abbreviations: AFP, alpha-fetoprotein; AFP-L3%, lens culinaris-agglutinin-reactive fraction of AFP; DCP, des-gamma-carboxy prothrombin.

**FIGURE 2:**
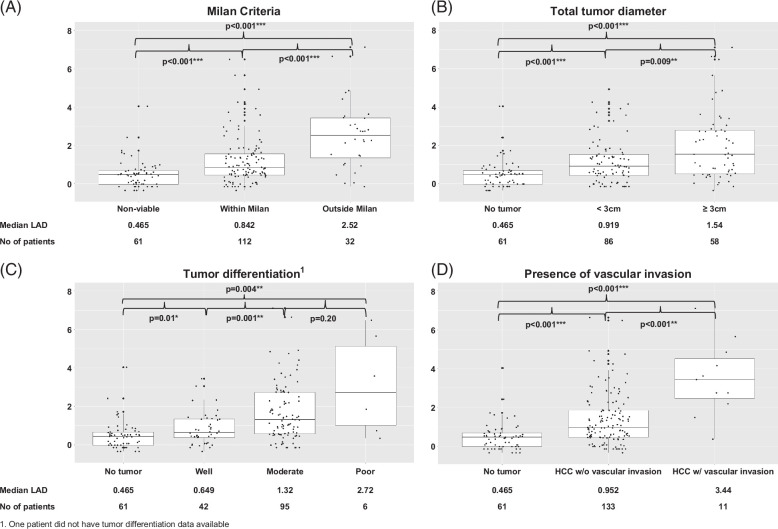
Median LAD score of derivation cohort stratified by explant histology. (A) Milan criteria (bars represent 25th and 75th percentiles, vertical lines extending out from the box represent 1.5 [Quartile 3 − Quartile 1], bold line inside the box plot represents median levels). Level of significance: *p* < 0.01 (Wilcoxon test). (B) Total tumor diameter (bars represent 25th and 75th percentiles, vertical lines extending out from the box represent 1.5 [Quartile 3 − Quartile 1], bold line inside the box plot represents median levels). Level of significance: *p* < 0.01 (Wilcoxon test). (C) Tumor differentiation^1^ (bars represent 25th and 75th percentiles, vertical lines extending out from the box represent 1.5 [Quartile 3 − Quartile 1], bold line inside the box plot represents median levels). Level of significance: *p* < 0.01 (Wilcoxon test). (D) Presence of vascular invasion (bars represent 25th and 75th percentiles, vertical lines extending out from the box represent 1.5 [Quartile 3 − Quartile 1], bold line inside the box plot represents median levels). Level of significance: *p* < 0.001 (Wilcoxon test). ^1^One patient did not have tumor differentiation data available.

The median LAD score was significantly higher in individuals with viable tumors compared to those with nonviable tumors on explant (1.06 vs. 0.465, respectively; *p* < 0.001). The LAD score correlates with the extent of the tumor on explant based on Milan criteria, cumulative total tumor diameter, tumor grade/differentiation, and the presence of microvascular/macrovascular invasion (Figures 2A–D; note that comparative analysis of poorly differentiated grade is limited by a small sample size).

### LAD score performance for posttreatment tumor detection

The final model using 3 tumor markers had an AUROC of 0.736 (95% CI: 0.666, 0.806). The optimal cutoff for the LAD score was 0.927 and yielded a sensitivity of 55.6% and a specificity of 85.1%. AUROC of the LAD score was greater than that of AFP alone (0.736 vs. 0.655, *p* = 0.009). The LAD score had a greater AUROC than imaging (0.736 vs. 0.643, *p* = 0.045) and had a trend toward a higher sensitivity at a fixed specificity of 82.0% (0.569 [95% CI: 0.489, 0.650] vs. 0.465 [95% CI: 0.384, 0.547]). In etiology stratified analysis, AUROC of the LAD score was superior to imaging in patients with Hepatitis C (*p* < 0.001); the LAD score also had a higher AUROC among patients with MASH, although this difference did not reach statistical significance (Table 3). Overall results were consistent when patients were stratified based on demographic variables (data not shown).

**TABLE 3:** Optimal cutoff and accuracy for LAD score and individual biomarkers, stratified by the etiology of liver disease

Etiology of liver disease	Optimal cutoff	Sensitivity (95% CI)	Specificity (95% CI)	AUROC (95% CI)	*p* ^a^ (AUROC)
Hepatitis C (n = 102)					
Imaging		0.437 (0.324, 0.549)	0.742 (0.581, 0.903)	0.589 (0.492, 0.687)	Reference
AFP (ng/mL)	13.5	0.394 (0.282, 0.507)	0.871 (0.742, 0.968)	0.648 (0.539, 0.758)	0.43
AFP-L3% (%)	5.05	0.747 (0.648, 0.845)	0.548 (0.387, 0.710)	0.654 (0.544, 0.764)	0.36
DCP (ng/mL)	1.05	0.662 (0.549, 0.761)	0.839 (0.710, 0.968)	0.766 (0.685, 0.848)	**0.006**
LAD score	0.927	0.592 (0.479, 0.704)	0.871 (0.742, 0.967)	0.747 (0.653, 0.841)	**0.02**
Metabolic dysfunction–associated steatotic liver diseases (n = 29)					
Imaging		0.636 (0.455, 0.818)	0.857 (0.571, 1.000)	0.747 (0.573, 0.920)	Reference
AFP (ng/mL)	16.5	0.273 (0.091, 0.455)	1.000 (1.000, 1.000)	0.565 (0.329, 0.800)	0.16
AFP-L3% (%)	10.4	0.636 (0.455, 0.818)	0.857 (0.571, 1.000)	0.679 (0.482, 0.875)	0.48
DCP (ng/mL)	1.05	0.636 (0.455, 0.818)	1.000 (1.000, 1.000)	0.818 (0.715, 0.921)	0.49
LAD score	0.533	0.773 (0.591, 0.955)	0.857 (0.571, 1.000)	0.799 (0.634, 0.963)	0.61
Alcohol (n=29)					
Imaging		0.364 (0.182, 0.546)	0.857 (0.571, 1.000)	0.610 (0.437, 0.784)	Reference
AFP (ng/mL)	5.5	0.546 (0.364, 0.773)	1.000 (1.000, 1.000)	0.756 (0.584, 0.929)	0.24
AFP-L3% (%)	1.55	0.818 (0.636, 0.955)	0.571 (0.286, 0.857)	0.659 (0.400, 0.919)	0.78
DCP (ng/mL)	1.02	0.636 (0.409, 0.818)	0.571 (0.286, 0.857)	0.536 (0.255, 0.817)	0.69
LAD score	0.475	0.773 (0.591, 0.955)	0.571 (0.143, 0.857)	0.679 (0.413, 0.945)	0.70
Hepatitis B (n = 24)					
Imaging		0.615 (0.308, 0.846)	1.000 (1.000, 1.000)	0.808 (0.670, 0.945)	Reference
AFP (ng/mL)	6.5	0.539 (0.231, 0.769)	1.000 (1.000, 1.000)	0.755 (0.559, 0.951)	0.64
AFP-L3% (%)	3.9	0.539 (0.308, 0.769)	0.909 (0.727, 1.000)	0.734 (0.572, 0.897)	0.46
DCP (ng/mL)	1.1	0.385 (0.154, 0.692)	0.909 (0.727, 1.000)	0.636 (0.465, 0.807)	**0.04**
LAD score	0.618	0.615 (0.385, 0.846)	0.909 (0.727, 1.000)	0.731 (0.520, 0.941)	0.49
Others (n = 21)					
Imaging		0.375 (0.188, 0.625)	0.800 (0.400, 1.000)	0.588 (0.356, 0.819)	Reference
AFP (ng/mL)	12.5	0.813 (0.625, 1.000)	0.400 (0.000, 0.800)	0.525 (0.215, 0.835)	0.65
AFP-L3% (%)	7.15	0.813 (0.625, 1.000)	0.600 (0.200, 1.000)	0.681 (0.415, 0.947)	0.65
DCP (ng/mL)	1.9	0.500 (0.250, 0.750)	0.800 (0.400, 1.000)	0.594 (0.305, 0.882)	0.98
LAD score	0.916	0.500 (0.250, 0.750)	0.800 (0.400, 1.000)	0.588 (0.293, 0.882)	0.46
All (n = 205)					
Imaging	17	0.465 (0.382, 0.549)	0.819 (0.721, 0.918)	0.643 (0.579, 0.706)	Reference
AFP (ng/mL)	5.05	0.306 (0.236, 0.382)	0.951 (0.885, 1.000)	0.655 (0.578, 0.732)	0.80
AFP-L3% (%)	1.02	0.736 (0.660, 0.806)	0.574 (0.459, 0.705)	0.679 (0.604, 0.754)	0.45
DCP (ng/mL)	0.927	0.618 (0.542, 0.694)	0.820 (0.721, 0.918)	0.716 (0.649, 0.783)	0.13
LAD score		0.556 (0.472, 0.639)	0.851 (0.754, 0.934)	0.736 (0.666, 0.806)	**0.045**

Significant (*p* < 0.01) values are in bold.

a *p* value for comparison of AUROC between imaging and individual biomarkers/LAD score.

Abbreviations: AFP, alpha-fetoprotein; AFP-L3%, lens culinaris-agglutinin-reactive fraction of AFP; DCP, des-gamma-carboxy prothrombin.

In patients with viable tumors on imaging (n = 78), 67 (85.9%) had viable tumors on explant (Supplemental Table S2, http://links.lww.com/HC9/A927). Among patients with no viable tumor on imaging (n = 127), 77 (60.6%) had viable tumors on explant. In this subgroup of patients, the median LAD score was higher in those with viable versus nonviable tumors on explant (1.05 vs. 0.43, respectively; *p* < 0.001). The LAD score had an AUROC of 0.764 (95% CI: 0.648, 0.823) for the detection of viable tumor on explant with a sensitivity of 55.8% and a specificity of 88.0% at the cutoff of 0.927.

In patients with small (<2 cm in diameter) tumors (N = 100), the LAD score was superior to imaging in predicting tumor viability on explant (AUROC 0.721 vs. 0.595, *p* = 0.02). The LAD score and imaging performed similarly for detecting viable tumors ≥2 cm (AUROC 0.771 vs. 0.751, *p* = 0.75).

In addition, 38 (18.5%) individuals had a tumor on explant that was either outside Milan criteria and/or had microvascular invasion. In this group, 31 individuals (81.6%) had a LAD score greater than the cutoff of 0.927. Fourteen out of 38 (36.8%) individuals had a nonviable tumor on imaging; among these patients, 12 (85.7%) had positive LAD scores.

In an explorative analysis, a predictive model using the 3 biomarkers and cross-sectional imaging was calculated using a multivariate generalized linear model (Table 4), yielding an AUROC of 0.777 (0.710, 0.845). The biomarker and imaging model had a sensitivity of 69.4% and a specificity of 78.7% at the optimal cutoff of 0.761.

**TABLE 4:** AUROC comparison between the LAD score, individual biomarkers, and imaging for predicting HCC viability

		Derivation cohort (n = 205)			Validation cohort^a^ (n = 117)		
Predictor	Optimal cutoff	AUROC (95% CI)	Sensitivity (%)	Specificity (%)	AUROC (95% CI)	Sensitivity (%)	Specificity (%)
LAD score	0.927	0.736 (0.666, 0.806)	55.6	85.1	0.832 (0.753, 0.911)	72.5	89.4
AFP (ng/mL)	17	0.655 (0.578, 0.732)	30.6	95.1	0.718 (0.624, 0.812)	39.2	89.4
AFP-L3% (%)	5.05	0.679 (0.604, 0.754)	73.6	57.4	0.746 (0.656 0.837)	82.4	54.5
DCP (ng/mL)	1.02	0.716 (0.649, 0.783)	61.8	82.0	0.768 (0.686, 0.850)	68.6	78.8
Imaging^b^	NA	0.643 (0.579, 0.706)	46.5	81.9	0.667 (0.581, 0.753)	33	100
LAD score + imaging^b^	0.761	0.777 (0.710, 0.845)	69.4	78.7	0.888 (0.771–1.00)	56.7	100

a Sensitivity and specificity of the validation cohort were obtained using the optimal cutoff calculated from the derivation cohort.

b AUROC and sensitivity/specificity for the “Imaging” and “LAD + Imaging” predictors for the validation cohort were obtained using the subset of patients with explant histology (n = 38).

Abbreviations: AFP, alpha-fetoprotein; AFP-L3%, lens culinaris-agglutinin-reactive fraction of AFP; DCP, des-gamma-carboxy prothrombin.

### Model validation

Among the 117 patients, 51 (43.6%) posttreatment cases had evidence of viable tumors on initial surveillance imaging; among cases with viable tumors, only 26 (51.0%) had AFP levels ≥10. Patients treated with ablation were more likely to have nonviable tumor posttreatment compared to those treated with embolization (68.0% vs. 53.3%), although this difference did not reach statistical significance (*p* = 0.19). Tumor viability was assessed with cross-sectional imaging for most patients as explant histology was not available. Sensitivity analysis was performed on patients who underwent liver transplant using histology to assess tumor viability: 40 patients underwent liver transplant, 38 of whom had biomarkers and imaging obtained <3 months before the transplant. Median AFP, AFP-L3%, and DCP levels were all higher in individuals with viable tumors after treatment compared to nonviable (Figure 1). A similar pattern was seen with the median LAD score (1.68 in viable tumors vs. 0.45 in nonviable tumors, *p* < 0.001).

The model had an AUROC of 0.832 (95% CI: 0.753, 0.911), a sensitivity of 72.5%, and a specificity of 89.4% using the optimal cutoff of 0.927, which was selected from the derivation set (Table 4). Sensitivity analysis was performed by excluding 12 (10.1%) individuals with triple-negative pretreatment biomarkers—in this cohort, the AUROC was 0.853 (95% CI: 0.775, 0.931) with a sensitivity of 74.5% and a specificity of 91.3% using the optimal cutoff of 0.927. There was no statistically significant difference in demographics, tumor size, or the number of tumors between the 12 biomarker-negative patients and the rest of the cohort (Supplemental Table S3, http://links.lww.com/HC9/A927). Given the small number of patients in the validation data set, only limited subgroup analysis could be performed based on HCC etiology and treatment modality. The AUROC of the LAD score was higher in patients with nonviral etiology of HCC compared to viral (0.890 vs. 0.759) and higher for those treated with TACE compared to transarterial radioembolization (0.900 vs. 0.799); however, neither comparative difference reached statistical significance (*p* = 0.12 and *p* = 0.23, respectively).

Among the 38 (32.5%) patients who underwent liver transplants and were included in the histology sensitivity analysis, 30 had viable tumors on explant. In this subgroup, the AUROC of the LAD score was 0.844 (95% CI: 0.701, 0.99). The LAD score performed better than cross-sectional imaging (AUROC 0.667 [95% CI: 0.581, 0.753]) (*p* = 0.03). When the aforementioned model incorporating LAD score and cross-sectional imaging was applied to this subgroup, the AUROC improved to 0.888 (95% CI: 0.771–1.00; Table 4).

## DISCUSSION

Given the increasing utilization of locoregional therapy for HCC, accurate posttreatment surveillance modalities are becoming ever more crucial.^9,26,27^ We derived the LAD score using biomarkers obtained from a large single-institution cohort and found that the score is higher in patients with HCC and positive pretreatment biomarkers who have viable tumors after treatment than in those with nonviable tumors. Surprisingly, the accuracy of the LAD score was higher than the accuracy of cross-sectional images with explant histology as a gold standard, highlighting the utility of the LAD score in the treatment response assessment after locoregional treatment. Moreover, the LAD score showed greater accuracy in detecting tumors <2 cm in diameter than imaging and was more accurate for tumors that were outside the Milan criteria and/or microvascular invasion, which is associated with a greater risk of tumor recurrence.^32^ The LAD score has excellent performance for the detection of viable tumors among patients with no viable tumors on imaging assessment. The accuracy of the LAD score for detecting viable HCC was validated on an independent data set, in which imaging was used primarily to determine HCC viability. The LAD score retained its high sensitivity and specificity in the validation set, suggesting that it may be useful in the clinical setting where imaging (rather than histology) is used to determine tumor viability.

Locoregional treatment is a standard treatment in patients with intermediate-stage HCC.^33^ Following liver-directed cancer therapy, patients undergo imaging to evaluate treatment response, which is essential for ongoing management. However, assessing treatment response can often be challenging due to treatment-related nonspecific changes in imaging, particularly after locoregional treatment, and definitive assessment can only be made after repeated cross-sectional images with longer follow-up. While the LI-RADS criteria is accurate for diagnosing untreated HCC (94% accuracy for LR-5 lesions), prior studies show poor radiologic-histological correlation of HCC viability after local ablation or TACE with discordant results seen in 38% of patients.^34,35^ Serum AFP has been shown to complement imaging-based treatment response assessment—a previous study showed that changes in AFP can serve as a marker for posttreatment response assessment in patients with HCC with elevated pretreatment AFP levels.^36,37^ However, less than half of all patients with HCC have elevated AFP, limiting its utility for patients who have normal pretreatment AFP levels. Hence, developing a highly accurate biomarker for posttreatment response assessment of HCC, particularly after locoregional treatment is an urgent clinical unmet need.

In the current study, we developed the LAD score using a triple serum tumor marker for HCC, which maintained its accuracy among the different etiologies of liver disease in exploratory analysis, suggesting that it will retain its predictive utility. Prior studies have found that the GALAD score has good accuracy for detecting HCC across different etiologies, and our preliminary results suggest that the AUROC in cases of HCV and MASH may be greater.^19^ Definitive conclusions on the etiology-specific performance of the LAD score could not be made due to the small sample size and a larger ongoing prospective study will confirm our results.

The accuracy of the LAD score was preserved in the subgroup of imaging-negative patients, suggesting that it may be a useful adjunct to imaging for posttreatment surveillance. Notably, we found that the LAD score outperformed cross-sectional imaging in detecting small HCC tumors (<2 cm in diameter) and performed similarly in detecting larger tumors, which indicates that the LAD score may be helpful for early tumor detection. Upon validation in a larger prospective study, the LAD score may have clinical utility as a risk stratification tool—for example, patients with an elevated LAD score after locoregional treatment but have nonviable or indeterminate posttreatment imaging results should be considered for short-term surveillance (ie, imaging every 1–2 months instead of 3–4 months) or alternative imaging modalities (ie, CT instead of MRI or vice versa). Conversely, extending surveillance duration in patients with a negative LAD score and negative imaging may help reduce health care burden and costs; however, prospective longitudinal data are needed for larger-scale validation, which should include cost-effectiveness analysis of LAD score for the assessment of posttreatment tumor viability.

Currently, AFP is used in a similar manner for risk stratification; however, incorporating multiple biomarkers expands the utility of this score to non-AFP–producing tumors. Moreover, our analysis showed that the AUROC of the LAD score is significantly greater than that of AFP alone. Multiple studies have demonstrated poor adherence to imaging-based HCC surveillance, and this problem will likely be exacerbated by the rising demand and limited availability of imaging.^38,39^ Serum-based biomarker tests are more easily accessible to patients and may decrease the need for frequent use of cross-sectional images. Among patients who have cross-sectional imaging and biomarkers test concurrently, our results suggest that a combined model might have a higher accuracy—when cross-sectional imaging results were incorporated into the LAD model, the AUROC improved to nearly 0.8. Additional studies with a larger sample size are needed to confirm these results.

Our study has several strengths. To our knowledge, this is the first study using a biomarker-derived model to assess posttreatment viable tumors. All patients in the derivation cohort had explant histology to confirm the presence or absence of viable tumors. The initial model was validated on an independent data set and performed similarly for the detection of viable tumors.

There are also some limitations to our study. First, we excluded patients with pretreatment-negative biomarkers for model derivation. This was intended as tumor marker level will likely have a minimal role for posttreatment response assessment among those with normal tumor marker levels before cancer treatment. This would allow us to develop a model with maximum accuracy in treatment response assessment. However, excluding individuals with negative pretreatment biomarkers limits the applicability of our model in detecting aggressive tumors that may change from biomarker negative to positive. Although the model was developed for those with pretreatment tumor marker elevation, the LAD model still performed well in the validation cohort, which included pretreatment biomarker-negative cases. Given the relatively small size of the validation data set, larger-scale validation is needed for broader application of LAD score in routine clinical practice including for those with negative tumor markers before cancer treatment. Second, while most demographic features did not differ significantly between our 2 study cohorts, there are still likely unmeasured differences between the 2 groups because the derivation cohort contained only patients who had undergone liver transplants. Third, while the validation cohort was intended to represent the general population of patients with HCC, accurate representation may be limited due to the relatively small sample size and retrospective nature of the study. Therefore, larger prospective biomarker studies are needed to confirm our results. Finally, only a small subset of patients in the validation set had explant data; thus, tumor viability was determined based on the combination of radiologic and pathologic assessment. This suggests that the LAD score maintains its accuracy in the clinical setting (where imaging rather than histology is generally used to assess for tumor viability); additionally, the performance of the LAD model was excellent in the subset of the validation cohort with explant data. Nevertheless, validating the model on a cohort that had histologic data for all patients would have provided greater support for its accuracy.

## CONCLUSIONS

In patients with serum biomarker-positive HCC, the LAD score has utility for assessing posttreatment response and can supplement cross-sectional imaging. Prospective studies with a larger sample size are needed to investigate the accuracy of LAD score for posttreatment response assessment, explore its utility for recurrence monitoring, and further develop and validate a biomarker and imaging model.

## Supplementary Material

SUPPLEMENTARY MATERIAL
