# Supplementary material for: Development and validation of a biomarker index for HCC treatment response
Source: Hepatol Commun. 2024 Jun 19;8(7):e0466. doi: 10.1097/HC9.0000000000000466 (PMC11186807; doi:10.1097/HC9.0000000000000466)
Supplement: SUPPLEMENTARY MATERIAL [file hc9-8-e0466-s001.docx]

Supplemental Figure 1: Patient selection flowchart


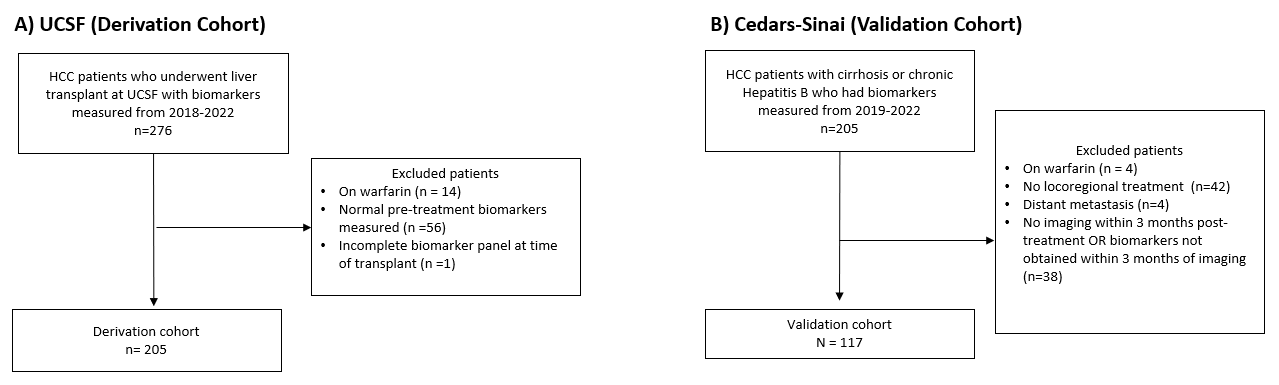


Supplemental Table 1: Comparison of demographic features between pre-treatment biomarker negative and biomarker positive patients in the **derivation cohort**

| **Category** | **Biomarker positive (n=205)** | **Biomarker negative (n=56)** | **p-value** |
| --- | --- | --- | --- |
| **Age**  < 65 years  ≥ 65 years | 108 (52.7%)  97 (47.3%) | 35 (62.5%)  21 (37.5%) | 0.23 |
| **Sex**  Male  Female | 148 (72.2%)  57 (27.8%) | 46 (82.1%)  10 (17.9%) | 0.17 |
| **Race**  White (non-Hispanic)  Hispanic  Asian  Black/other | 83 (40.5%)  65 (31.7%)  41 (20.0%)  16 (7.80%) | 28 (50.0%)  11 (19.6%)  13 (23.2%)  4 (7.1%) | 0.33 |
| **Etiology of liver disease**  Hepatitis C  Metabolic dysfunction-associated steatohepatitis  Alcohol  Hepatitis B  Other/Unknown | 102 (49.8%)  29 (14.1%)  29 (14.1%)  24 (11.7%)  21 (10.2%) | 22 (39.3%)  6 (10.7%)  7 (12.5%)  12 (21.4%)  9 (16.1%) | 0.23 |
| **Initial tumor burden**  **(25-75^th^ percentile)**  Median initial number of tumors  Median initial largest tumor size (cm) | 1.00 (1.00, 2.00)  2.50 (2.10, 3.30) | 1.00 (1.00, 1.25)  2.50 (2.10, 3.25) | 0.41  0.74 |

Supplementary Table 2: Biomarker, imaging (pre-transplant) and histology for patients with hepatocellular carcinoma and available explant data

| **Category** | **Derivation cohort**  **N = 205** | | **Validation cohort**  **N = 38** | |
| --- | --- | --- | --- | --- |
|  | Viable (N = 144) | Non-viable (N=61) | Viable (N=30) | Non-viable (N=8) |
| **Imaging** | | | | |
| **LR-TR viable on imaging**  Yes  No | 67 (46.5%)  77 (53.5%) | 11 (18.0%)  50 (82.0%) | 10 (33.3%)  20 (66.7%) | 0 (0.0%)  8 (100%) |
| **Histology^1^** | | | | |
| **Number of tumors**  ≤ 3  > 3 | 117 (81.2%)  27 (18.8%) | | 20 (66.7%)  10 (33.3%) | |
| **Median tumor size (cm)**  **(25^th^-75^th^ percentile)**  Largest tumor diameter  Cumulative tumor diameter | 1.6 (1.1, 2.1)  2.4 (1.3, 3.9) | | 2.05 (1.25, 3.00)  3.8 (1.7, 5.48) | |
| **Tumor Grade^2^**  Well differentiated  Moderately differentiated  Poorly differentiated | 42 (29.2%)  95 (66.0%)  6 (4.2%) | | 3 (10.0%)  23 (76.7%)  4 (13.3%) | |

1. Only includes patients with viable tumor on explant
2. For individuals with multiple viable tumors, only the highest tumor grade was counted

Supplemental Table 3: Comparison of demographic features between pre-treatment biomarker negative and biomarker positive patients in the **validation cohort**

| **Category** | **Biomarker positive (n=105)** | **Biomarker negative (n=12)** | **p-value** |
| --- | --- | --- | --- |
| **Age**  < 65 years  ≥ 65 years | 35 (33.3%)  70 (66.7%) | 4 (33.3%)  8 (66.7%) | 1 |
| **Sex**  Male  Female | 70 (66.7%)  35 (66.7%) | 9 (75.0%)  3 (25.0%) | 0.56 |
| **Race**  White (non-Hispanic)  Hispanic  Asian  Black/other | 34 (32.4%)  46 (43.8%)  19 (18.1%)  6 (5.7%) | 5 (41.7%)  6 (50.0%)  0 (0.0%)  1 (8.3%) | 0.81 |
| **Etiology of liver disease**  Hepatitis C  Metabolic dysfunction -associated steatohepatitis  Alcohol  Hepatitis B  Other/Unknown | 36 (34.3%)  23 (21.9%)  18 (16.8%)  17 (17.1%)  11 (10.5%) | 6 (50.0%)  3 (25.0%)  3 (25.0%)  0 (0.0%)  0 (0.0%) | 0.88 |
| **Initial tumor burden**  **(25-75^th^ percentile)**  Median initial number of tumors  Median initial largest tumor size (cm) | 1.0 (1.0, 2.0)  2.7 (2.0, 4.0) | 2.0 (1.0, 2.8)  2.5 (2.0, 4.1) | 0.11  0.77 |
